# Supplementary material for: Body mass index trends and its impact of under and overweight on outcome among PLHIV on antiretroviral treatment in rural Tanzania: A prospective cohort study
Source: PLoS One. 2023 Aug 22;18(8):e0290445. doi: 10.1371/journal.pone.0290445 (PMC10443839; doi:10.1371/journal.pone.0290445)
Supplement: S1 Table — (PDF) [file pone.0290445.s001.pdf]

S1 Table. Patients' characteristics at ART initiation by the availability of follow-up BMI measurements (numbers with their respective percentages)

Characteristics	Follow-up BMI measurements available		P-value
	No	Yes	
Overall	154 (7.2)	1975 (92.8)	
Sex			
Male	70 (45.5)	810 (41.0)	0.28
Female	84 (54.6)	1165 (59.0)	
Age in years			
19 - 34	52 (33.8)	579 (29.3)	0.50
35 - 44	54 (35.1)	725 (36.7)	
45 and above	48 (31.2)	671 (34.0)	
Occupation			
Non-farmers	24 (15.6)	278 (14.1)	0.61
Farmers	130 (84.4)	1697 (85.9)	
Education			
No education	19 (12.3)	210 (10.6)	0.76
Primary school	125 (81.2)	1648 (83.4)	
Above primary school	10 (6.5)	117 (5.9)	
ART Line			
First line[a]	150 (97.4)	1960 (99.2)	0.04
Second line[b]	4 (2.6)	15 (0.8)	
CD4 count, cells/µL			
Below 100	47 (35.1)	486 (27.4)	0.22
100 - 199	21 (15.7)	369 (20.8)	
200 - 349	34 (25.4)	469 (26.5)	
350 and above	32 (23.9)	448 (25.3)	
WHO stage			
Stage 1/2	68 (44.4)	1003 (51.6)	0.09
Stage 3/4	85 (55.6)	940 (48.4)	
Tuberculosis			
Negative	126 (83.4)	1549 (80.9)	0.45
Positive	25 (16.6)	365 (19.1)	
Hypertension			
No arterial hypertension	123 (95.4)	1774 (91.2)	0.10
Arterial hypertension	6 (4.7)	171 (8.8)	
Anemia status			
No anemic	37 (26.1)	620 (35.4)	0.02
Anemic	105 (73.9)	1132 (64.6)	

All participants had BMI measurement captured at ART initiation, as per the inclusion criteria for this study.

Percentages are of columns, except for the overall percentages (row percentage of all participants).

Chi-Square tests and Fisher's exact (if 25% of the cell count has frequency <5) p-values.

[a]First-line ART was AZT+3TC+NVP, AZT+3TC+EFV, TDF+FTC+EFV, TDF+FTC+NVP, TDF+3TC+EFV, ABC+3TC+EFV.
[b]Second-line ART was TDF+FTC+LPV/r, AZT+3TC+LPV/r, TDF+FTC+ATV/r.
