# Supplementary material for: Body mass index trends and its impact of under and overweight on outcome among PLHIV on antiretroviral treatment in rural Tanzania: A prospective cohort study
Source: PLoS One. 2023 Aug 22;18(8):e0290445. doi: 10.1371/journal.pone.0290445 (PMC10443839; doi:10.1371/journal.pone.0290445)
Supplement: S2 Table — (PDF) [file pone.0290445.s002.pdf]

S2 Table: Assessment of different change points

Time points (per 3 months)	Normal weight		Underweight		Overweight/obese	
	Estimates [95% Confidence interval]	P-value	Estimates [95% Confidence interval]	P-value	Estimates [95% Confidence interval]	P-value
Before until 6 months	1.009 [1.008, 1.011]	<.0001	1.023 [1.02, 1.027]	<.0001	1.005 [1.002, 1.008]	0.001
After 6 months	0.999 [0.997, 1.000]	0.070	0.997 [0.995, 1.000]	0.023	1.000 [0.997, 1.003]	0.935
Before until 7 months	1.008 [1.007, 1.010]	<.0001	1.020 [1.017, 1.023]	<.0001	1.005 [1.002, 1.008]	0.001
After 7 months	0.999 [0.997, 1.000]	0.060	0.997 [0.995, 0.999]	0.011	1.000 [0.997, 1.003]	0.903
Before until 8 months	1.007 [1.006, 1.009]	<.0001	1.017 [1.015, 1.020]	<.0001	1.004 [1.002, 1.007]	0.001
After 8 months	0.998 [0.997, 1.000]	0.06	0.996 [0.994, 0.999]	0.006	1.000 [0.996, 1.003]	0.836
**Before until 9 months**	**1.007 [1.005, 1.008]**	**<.0001**	**1.016 [1.013, 1.018]**	**<.0001**	**1.004 [1.002, 1.006]**	**0.001**
**After 9 months**	**0.998 [0.996, 1.000]**	**0.047**	**0.996 [0.993, 0.999]**	**0.003**	**1.000 [0.996, 1.003]**	**0.842**
Before until 12 months	1.005 [1.004, 1.007]	<.0001	1.012 [1.010, 1.014]	<.0001	1.004 [1.002, 1.006]	0.001
After 12 months	0.996 [0.994, 0.999]	0.005	0.995 [0.992, 0.998]	0.002	0.998 [0.993, 1.004]	0.571

Estimates based on an adjusted stratified linear mixed model and time modeled using piecewise regression, among patients with non-missing data at ART initiation.

Empirical standard error was used.

The BMI outcome was transformed using a natural logarithm, and the estimates should be interpreted in terms of the percent change of actual BMI.

All analyses performed for different change points were performed in multivariable models using covariates for the main analysis.
