# Supplementary material for: Body mass index trends and its impact of under and overweight on outcome among PLHIV on antiretroviral treatment in rural Tanzania: A prospective cohort study
Source: PLoS One. 2023 Aug 22;18(8):e0290445. doi: 10.1371/journal.pone.0290445 (PMC10443839; doi:10.1371/journal.pone.0290445)
Supplement: S3 Table — (PDF) [file pone.0290445.s003.pdf]

S3 Table: Patient characteristics at ART initiation by outcomes (numbers with their respective percentages)

Characteristics	In care	Death	Loss to follow-up	Transfer out	Total
Overall	1202 (56.5)	107 (5.0)	592 (27.8)	228 (10.7)	2129 (100)
Follow-up time in months, median (IQR)	38 (21-53)	3 (1-10)	8 (5-21)	7 (1-23)	22 (8-44)
Body Mass Index, kg/m^2					
Normal	775 (64.5)	62 (57.9)	393 (66.4)	145 (63.6)	1375 (64.6)
Underweight	184 (15.3)	36 (33.6)	125 (21.1)	53 (23.3)	398 (18.7)
Overweight and Obese	243 (20.2)	9 (8.4)	74 (12.5)	30 (13.2)	356 (16.7)
Sex					
Male	475 (39.5)	52 (48.6)	271 (45.8)	82 (36.0)	880 (41.3)
Female	727 (60.5)	55 (51.4)	321 (54.2)	146 (64.0)	1249 (58.7)
Age in years					
19 - 34	321 (26.7)	30 (28.0)	200 (33.8)	80 (35.1)	631 (29.6)
35 - 44	469 (39.0)	33 (30.8)	198 (33.5)	79 (34.7)	779 (36.6)
45 and above	412 (34.3)	44 (41.1)	194 (32.8)	69 (30.3)	719 (33.8)
Occupation					
Non-farmers	172 (14.3)	15 (14.0)	85 (14.4)	30 (13.2)	302 (14.2)
Farmers	1030 (85.7)	92 (86.0)	507 (85.6)	198 (86.8)	1827 (85.8)
Education					
No education	112 (9.3)	13 (12.2)	73 (12.3)	31 (13.6)	229 (10.8)
Primary school	1023 (85.1)	87 (81.3)	481 (81.3)	182 (79.8)	1773 (83.3)
Above primary school	67 (5.6)	7 (6.5)	38 (6.4)	15 (6.6)	127 (6.0)
ART Line					
First line[a]	1194 (99.3)	106 (99.1)	583 (98.5)	227 (99.6)	2110 (99.1)
Second line[b]	8 (0.7)	1 (0.9)	9 (1.5)	1 (0.4)	19 (0.9)
CD4 count, cells/µL					
Below 100	262 (21.8)	56 (52.3)	156 (26.4)	59 (29.5)	533 (25.0)
100 - 199	229 (19.1)	12 (11.2)	106 (17.9)	43 (18.9)	390 (18.3)
200 - 349	304 (25.3)	14 (13.1)	132 (22.3)	53 (23.3)	503 (23.6)
350 and above	294 (24.5)	10 (9.4)	131 (22.1)	45 (19.7)	480 (22.6)

Missing data	113 (9.4)	15 (14.0)	67 (11.3)	28 (12.3)	223 (10.5)
WHO stage					
Stage 1/2	700 (58.2)	25 (23.4)	257 (43.4)	89 (39.0)	1071 (50.3)
Stage 3/4	485 (40.4)	81 (75.7)	322 (54.4)	137 (60.1)	1025 (48.1)
Missing data	17 (1.4)	1 (0.9)	13 (2.2)	2 (0.9)	33 (1.6)
Tuberculosis					
Negative	979 (81.5)	66 (61.7)	468 (79.1)	162 (71.1)	1675 (78.7)
Positive	191 (15.9)	37 (34.6)	105 (17.7)	57 (25.0)	390 (18.3)
Missing data	32 (2.7)	4 (3.7)	19 (3.2)	9 (4.0)	64 (3.0)
Hypertension					
No arterial hypertension	1073 (89.3)	88 (82.2)	530 (89.5)	206 (90.4)	1897 (89.1)
Arterial hypertension	112 (9.3)	10 (9.4)	37 (6.3)	18 (7.9)	177 (8.3)
Missing data	17 (1.4)	9 (8.4)	25 (4.2)	4 (1.8)	55 (2.6)
Anemia status					
Not anemic	435 (36.2)	13 (13.3)	153 (25.8)	56 (24.6)	657 (30.9)
Anemic	634 (52.8)	85 (79.4)	372 (62.8)	146 (64.0)	1237 (58.1)
Missing data	133 (11.1)	9 (8.4)	67 (11.3)	26 (11.4)	235 (11.0)

Column percentages are presented, percentages may be slightly below or above 100 due to rounding.

[a]First-line ART was AZT+3TC+NVP, AZT+3TC+EFV, TDF+FTC+EFV, TDF+FTC+NVP, TDF+3TC+EFV, ABC+3TC+EFV.

[b]Second-line ART was TDF+FTC+LPV/r, AZT+3TC+LPV/r, TDF+FTC+ATV/r.
